# The isoprenoid end product N6-isopentenyladenosine reduces inflammatory response through the inhibition of the NFκB and STAT3 pathways in cystic fibrosis cells

**DOI:** 10.1007/s00011-017-1123-6

**Published:** 2017-12-11

**Authors:** Antonietta Santoro, Elena Ciaglia, Vanessa Nicolin, Alessandra Pescatore, Lucia Prota, Mario Capunzo, Matilde V. Ursini, Stefania L. Nori, Maurizio Bifulco

**Affiliations:** 1 Department of Medicine, Surgery and Dentistry, “Scuola Medica Salernitana” University of Salerno, Via S. Allende, 84081 Baronissi, Salerno Italy; 2 Clinical Department of Medical, Surgical and Health Science, University of Trieste, Strada di Fiume 447, 34149 Trieste, Italy; 3 Institute of Genetics and Biophysics “Adriano Buzzati-Traverso” CNR, Via P. Castellino, 80131 Naples, Italy; 4 Department of Molecular Medicine and Medical Biotechnology, University of Naples “Federico II”, Via Pansini, 80131 Naples, Italy

**Keywords:** N6-isopentenyladenosine, Inflammation, NFκB, STAT3, Cystic fibrosis, Selenoproteins

## Abstract

**Objective:**

N6-isopentenyladenosine (iPA) is an intermediate of the mevalonate pathway that exhibits various anti-cancer effects. However, studies on its anti-inflammatory activity are scarce and underlying molecular mechanisms are unknown. Therefore, we aimed to investigate the ability of iPA to exert anti-inflammatory effects in the human cystic fibrosis (CF) cell model of exacerbated inflammation.

**Materials and methods:**

TNFα-stimulated CF cells CuFi-1 and its normal counterpart NuLi-1 were pre-treated with increasing concentrations of iPA and cell viability and proliferation were assessed by MTT and BrdU assays. The effect of iPA on IL-8 and RANTES secretion was determined by ELISA, and the activation and expression of signaling molecules and selenoproteins were studied by Western blot. To assess the direct effect of iPA on NFκB activity, luciferase assay was performed on TNFα-stimulated HEK293/T cells transfected with a NFκB reporter plasmid.

**Results:**

We demonstrated for the first time that iPA prevents IL-8 and RANTES release in TNFα-stimulated CF cells and this effect is mediated by increasing the expression of the direct NFκB inhibitor IκBα and decreasing the levels of STAT3. Consistent with this, we showed that iPA inhibited TNFα-mediated NFκB activation in HEK/293T cells. Finally, we also found that iPA improved the levels of glutathione peroxidase 1 and thioredoxin reductase 1 only in CF cells suggesting its ability to maintain sufficient expression of these anti-oxidant selenoproteins.

**Conclusions:**

Our findings indicate that iPA can exert anti-inflammatory activity especially in the cases of excessive inflammatory response as in CF.

## Introduction

N6-isopentenyladenosine (iPA) is a cytokinin originally identified in plants but also present in human cells in a free form or as a modified adenosine bound at position 37 of selenocysteine transfer RNA (Sec-tRNA) [1, 2]. The addition of iPA on adenosine is catalyzed by tRNA-isopentenyltransferase (tRNA-IPT) using dimethylallyl pyrophosphate (DMAPP)—an intermediate of the mevalonate pathway—as donor of the isoprenoid chain [3]. The tRNA[Ser]Sec is co-translationally inserted into selenoproteins at in-frame UGA codons and is required to improve fidelity and efficiency of the selenoprotein synthesis [4, 5]. In *Xenopus*, a site-specific mutation eliminating the site of isopentenylation in the tRNA[Ser]Sec gene markedly reduced the synthesis of the selenoproteins deiodinases [6], whereas in transgenic mice expressing iPA-deficient tRNA[Ser]Sec, a tissue-specific reduction of selenoprotein synthesis including glutathione peroxidase 1 (GPX1) and thioredoxin reductase 1 (TR1) was observed [7]. A number of evidence has shown that iPA induces antiproliferative and pro-apoptotic effects in a variety of tumors *in vitro* and *in vivo* even though its mechanism of action is not yet fully understood [8–10]. The existing data report that in human breast cancer cells, iPA-induced effects can be mediated by the inhibition of the Akt/NFκB cell survival pathway [11] and more recently it has been reported that iPA, phosphorylated by adenosine kinase (ADK) into 5′-iPA-monophosphate (iPAMP), is able to inhibit angiogenesis in vitro and in vivo, triggering the AMP-activated protein kinase (AMPK) [12]. However, only few studies reported that iPA has some immunomodulatory properties being able to selectively expand and directly target natural killer (NK) cells [13] and reduced mouse ear oedema in a murine model of croton oil-induced dermatitis [14]. These studies did not investigate in depth the effect of iPA in inflammatory response and no studies have ever investigated its anti-inflammatory activity in chronic inflammatory disease such as CF.

On the basis of the overall considerations, we aimed to ascertain the anti-inflammatory activity of iPA using a cystic fibrosis (CF) cell model. CF is well known to be a chronic inflammatory disease caused by mutations in the gene encoding the cystic fibrosis transmembrane conductance regulator (CFTR), an ATP-gated chloride channel which is expressed, among others, at the apical membrane of epithelial secretory cells of the airways. Loss of functional CFTR in airways promotes surface liquid depletion and defective mucociliary clearance producing a cruel circle of phlegm retention, infection and inflammation leading to pulmonary failure [15]. CFTR-deficient airway epithelial cells are characterized by an excessive inflammatory response and display signaling abnormalities, especially activation of nuclear factor-κB (NFκB) [16] leading to the overexpression of epithelial-derived cytokines and chemokines including the neutrophilic and macrophage chemoattractants IL-8 and RANTES [17, 18].

To study the effect of iPA on CF inflammation, we analyzed its ability to inhibit chemokine release from both CF and non-CF cells, stimulated or not with tumor necrosis factor α (TNFα) which is a key cytokine in the initiation of the early inflammatory process [19]. We used CuFi-1 cells derived from a human CF lung homozygous for the deletion of phenylalanine 508 in the CFTR protein (CFTR^ΔF508/ΔF508^), and its normal counterpart NuLi-1 (wild type). These non-cancerous cell models are reported to maintain the ion channel physiology and retained signal transduction responses to inflammatory stimuli expected for the genotypes [20]. Moreover, we also investigated the possible mechanism of action of iPA by analyzing NFκB, MAPK/ERK, and signal transducer and activator of transcription 3 (STAT3) signaling which are among the major pathways involved in CF inflammatory response [21, 22]. Finally, since it is known that anti-oxidant selenoproteins, such as glutathione peroxidases and thioredoxin reductases, are involved in inflammatory process [23, 24], we evaluated the effect of iPA on GPX1 and TR1 expression levels in both cell types.

## Materials and methods

### Drugs and drug treatment

N6-isopentenyladenosine (iPA) (Sigma Aldrich, St. Louis, MO, USA) was dissolved in DMSO and added to cell cultures at the indicated concentration and for the indicated time. 5-Iodotubercidin (5-Itu) was purchased from Tocris Bioscience (Bristol, UK), dissolved in ethanol and added to cell cultures at a concentration of 30 nM for 30 min before any other treatment. TNFα (R&D Systems, Minneapolis, MN, USA) was added at a concentration of 20 ng/ml (CuFi-1 and NuLi-1 cells) or 10 ng/ml (HEK 293/T cells) 1 h after any other treatment and left for 14 h.

### Cell cultures

Cystic fibrosis CuFi-1 cell line, derived from a CF human bronchial epithelium homozygous for the CFTR ΔF508 mutation (American Type Culture Collection, ATCC, Manassas, VA, USA) and non-CF cells NuLi-1 [20] were grown on human placental collagen type VI-coated flasks (Sigma Aldrich, Milan, Italy) in BEGM medium (Clonetics, Lonza, Walkersville, Inc). Human Embryonic Kidney (HEK) 293/T cells were cultured in Dulbecco’s modified Eagle’s medium (Invitrogen, Carlsbad, CA, USA) supplemented with 10% fetal bovine serum, 2 mM L-glutamine, penicillin (50 U/mL) and streptomycin (50 µg/mL). Cells were incubated at 37 °C in a humidified atmosphere at 5% CO_2_.

### Viability assay

Cell viability was analyzed using the MTT assay. Briefly, cells were seeded in 96-well plates at the density of 10^4^/well, left to adhere to the plate and then treated with increasing concentrations of iPA ranging from 0.1 to 10 μM for CuFi-1 and NuLi-1, and from 0.1 to 5 μM for HEK 293/T cells for 24 h and 48 h. 3-(4,5-Dimethylthiazol-2-yl)-2,5-diphenyl-tetrazolium bromide (MTT) was added (0.5 mg/ml final concentration) to each well and incubated at 37 °C for additional 4 h. Formazan products were dissolved in 10% Triton X-100, 0.1 N HCl in 2-propanol. Absorbance was determined at 595 nm using a microplate reader (Bio-Rad Laboratories srl, MI, Italy) as previously described [25].

### Proliferation assay

Cell proliferation was evaluated using a colorimetric bromodeoxyuridine (BrdU) cell proliferation ELISA kit (Roche Diagnostics, Milan, Italy). In brief, 10^4^ cells were seeded into 96-well plates and left to adhere to the plate. Cells were then treated with increasing concentrations of iPA (0.1 to 10 μM) for 24 and 48 h. BrdU (10 μM final concentration) was added for the last 16 h. After the incubation period, the medium was removed and the ELISA BrdU immunoassay was performed following manufacturer’s instructions. The colorimetric reaction was stopped with H_2_SO_4_, and the absorbance was measured at 450 nm using a microplate reader (Bio-Rad Laboratories, Milan, Italy) as previously described [26].

### Determination of IL-8 and RANTES release

For cytokine determinations, 1 × 10^6^ cells were plated and left to adhere to the plate. Then, cells were preincubated with iPA for 1 h and/or 5-Itu and then stimulated with TNFα for a further 14 h. Cultured media were then collected, centrifuged for 5 min at 2000 rpm and the production of IL-8 and RANTES was determined by enzyme-linked immunosorbent assay (ELISA) (R&D Systems) following manufacturer’s instructions. The used ELISAs were sensitive at 3.5, and 2 pg/ml, respectively. Cytokine concentration in cell-free media was calculated as pg/ml/10^6^ cells.

### Western blot analysis

Cells were collected by centrifugation, washed twice with PBS and resuspended in RIPA buffer (NaCl 150 mM, 1% triton X-100 pH 8.0, 0.5% sodium deoxycholate, 0.1% SDS, 50 mM Tris, pH 8.0) at 4 °C, then centrifuged at 13,000 rpm for 30 min. Supernatants were collected and protein concentrations were determined by Bio-Rad protein assay. Equal amounts of protein extracts (30 µg) were boiled in Laemmli’s buffer, fractioned on 12% SDS–PAGE and then transferred to nitrocellulose membranes (Amersham GE Healthcare, Milan, Italy). Membranes were blocked in TBS-T (50 mM Tris, 135 mM NaCl, and 5 mM KCl, 0,1% Tween-20) containing 5% non-fat dry milk, then incubated overnight at 4 °C with anti-phoshoERK 1/2, anti-phoshoSTAT3 (Tyr 705), anti-IKKα, anti-IKKβ, anti-IKKγ or anti-IkBα (all from Cell Signaling Technology Inc). To evaluate selenoprotein expression, anti-GPX1 and anti-TR1 (Abcam, Cambridge, UK) were also used. After three washes, blots were probed with mouse or rabbit horseradish peroxidase-conjugated secondary antibodies (Cell Signaling Technology) for 1 h at room temperature and then developed using the ECL chemiluminescence system (Amersham GE Healthcare). Finally, membranes were stripped and re-probed with total anti-ERK1/2, (Cell Signaling Technology) and anti α-tubulin used as loading controls (Abcam). Results are the mean of three independent experiments. Immunoreactive bands were quantified using Quantity One I-D analysis software (Bio-Rad).

### Cell transfection and NFκB activity

Transfection of HEK 293/T was carried out using CaPO_4_ method as already reported [26]. All transfections included the reporter plasmid (IgκB-Luc), an internal control TK-renilla, and a supplemental empty vector to maintain the total amount of transfected DNA constant in each cell culture. 24 h after transfection, cells were pre-treated with iPA (2.5–5 µM) for 1 h and then stimulated with TNFα. After 4 h, cells were lysed using a Luciferase Passive Lysis buffer (P/N E1941, from Promega Corporation). Cell lysates were then harvested and assayed using the Dual-Glo Luciferase Reporter Assay System (Promega). Luciferase activity was measured using a multiplate reader (GloMax^®^ 96 Microplate Luminometer, Promega), and values were normalized to the *Renilla* luciferase activity [27].

### Statistical analysis

Measurements were performed in triplicates, unless otherwise stated. Values were expressed as means of three independent experiments with three replicates each ± SD. Statistical differences between the treatments and the controls were evaluated by the Student’s *t* test. *P* values less than 0.05 were considered statistically significant.

## Results

### Cytotoxic activity of iPA in CF and non-CF cells

**Fig. 1:**
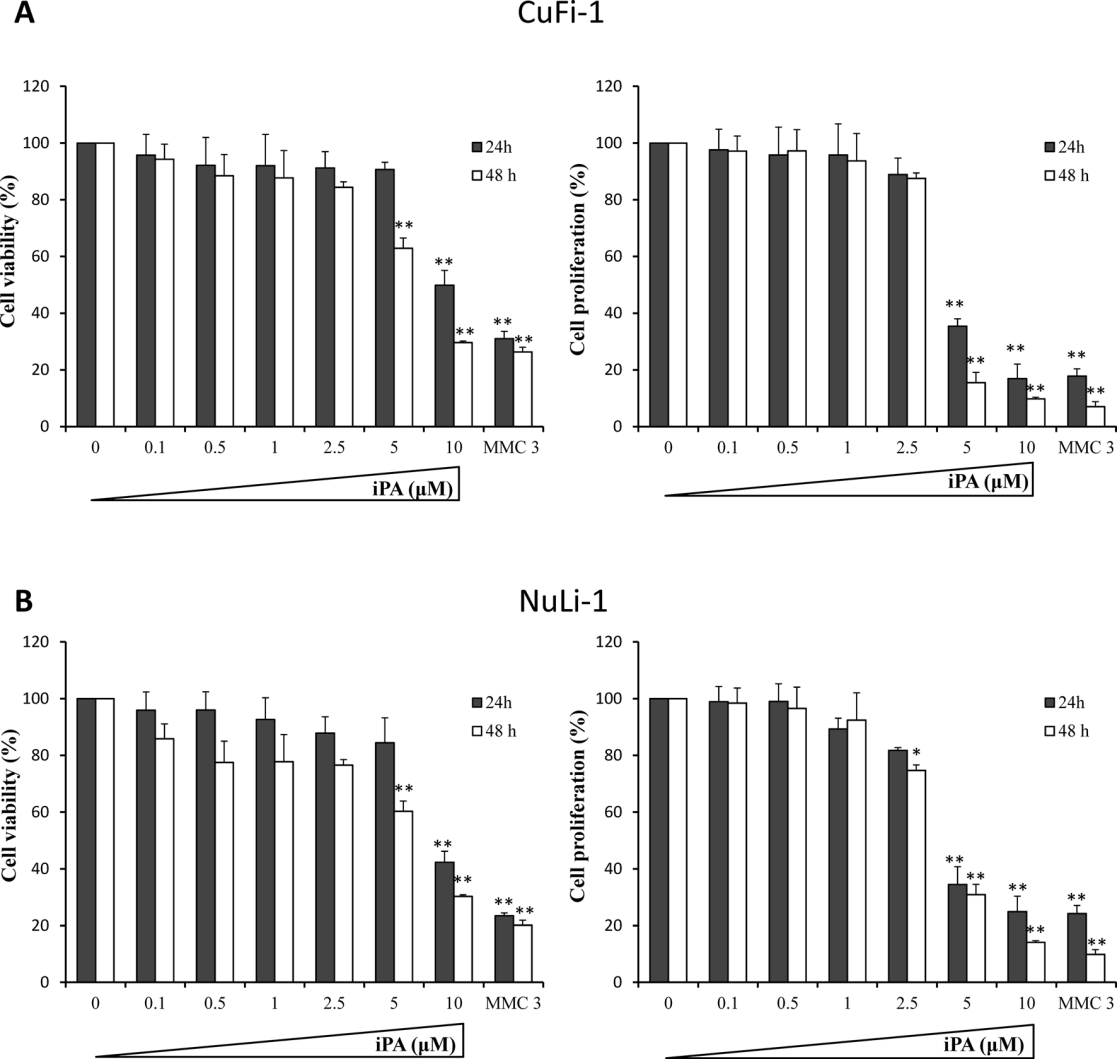
Effects of iPA on cell viability and proliferation in CuFi-1 and NuLi-1 cells. CuFi-1 (**a**) and NuLi-1 (**b**) cells were treated with increasing concentrations of iPA for 24 and 48 h. Cell viability was determined by MTT assay, whereas cell proliferation was detected using a BrdU incorporation assay (see materials and methods for details). Mytomycin C at a concentration of 3 µM (MMC 3) was used as positive control. Histograms report the percentage of viable and proliferating cells compared to controls (time zero, 100%). Data are shown as mean ± SD of three independent experiments each done in triplicates.*P < 0.05 and **P < 0.01 vs control

To determine the anti-inflammatory properties of iPA, first we investigated its cytotoxic activity in CF cells (CuFi-1) and non-CF cells (NuLi-1). Cells were treated with increasing concentrations of iPA from 0.1 to 10 µM for 24 and 48 h. MTT and BrdU assays were performed to evaluate cell viability and cell proliferation, respectively. Mitomycin C was used as a positive control. Figure 1 shows that iPA induced a general dose- and time-dependent inhibition of cell viability (left panels) and proliferation (right panels) in both CuFi-1 and NuLi-1 cells. Particularly iPA did not affect cell viability in the concentration range between 0.1 and 5 µM at 24 h of treatment in CuFi-1 cells (Fig. 1A), whereas it decreases cell viability of 37% at 5 µM and 48 h. CuFi-1 cell proliferation (Fig. 1A) was markedly inhibited by iPA (64% at 24 h and 5 µM), and at 10 µM only about 16% of cells were dividing. Concerning the results obtained in cell normal counterpart (NuLi-1, Fig. 1B), we observed a statistically significant reduction of cell growth starting from 2.5 µM concentration at 48 h of treatment, indicating that normal bronchial epithelial cells were slightly more susceptible to iPA-mediated inhibition of cell growth compared to CF cells. However, as in the case of CuFi-1 cells, iPA did not induce any significant cytotoxic effect until 5 µM at 24 h of treatment. Therefore, concentrations not exceeding 5.0 µM and a time-treatment below 24 h were chosen to study the potential anti-inflammatory effects of iPA.

### iPA selectively inhibits cytokine release in CF and non-CF cells

**Fig. 2:**
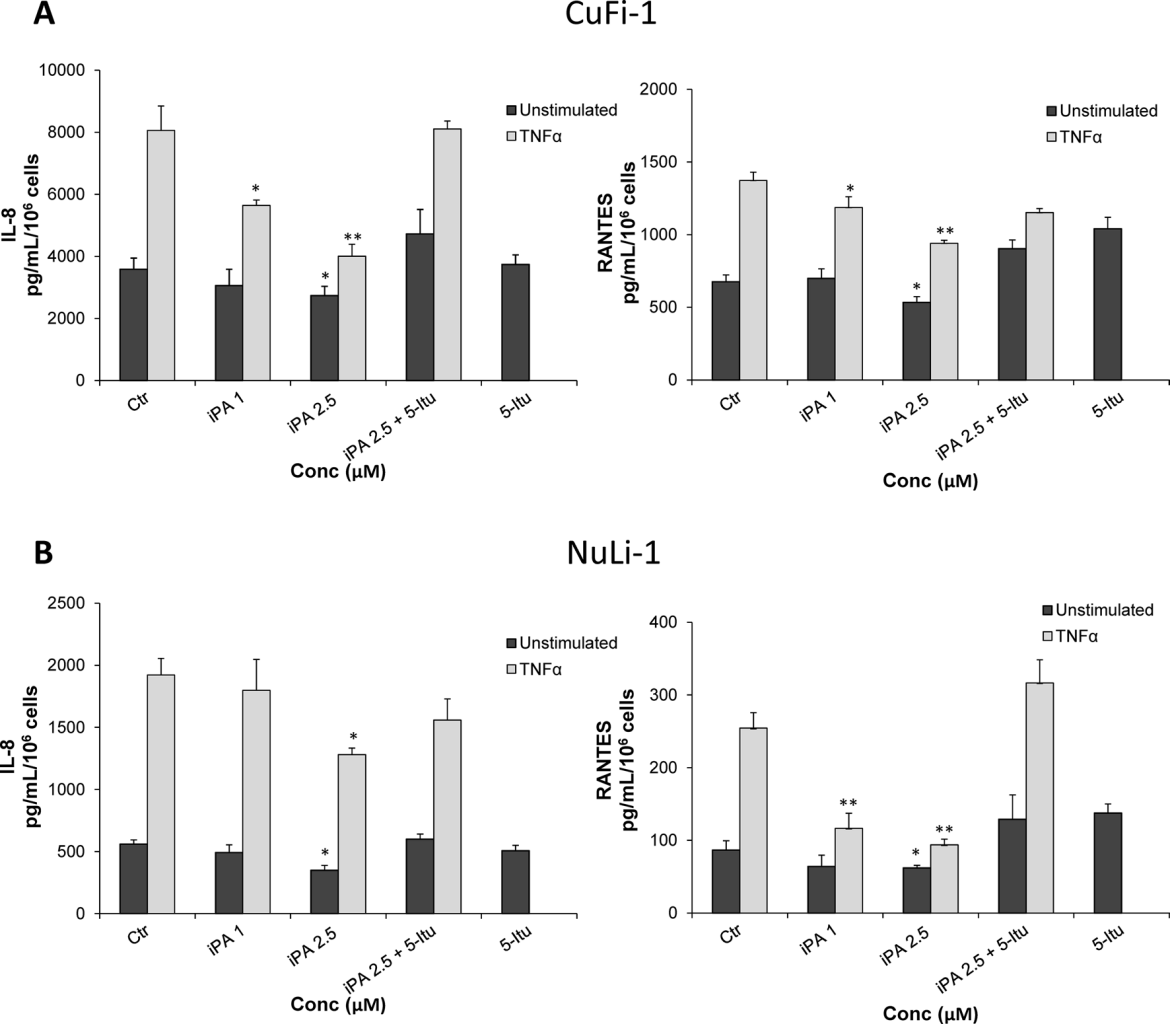
Effect of iPA on IL-8 and RANTES release in TNFα-stimulated and -unstimulated CuFi-1 and NuLi-1 cells. Cells were treated or untreated (controls, ctr) with iPA at indicated concentrations and then stimulated or not with TNFα as reported in Materials and Methods. IL-8 and RANTES levels were then detected in CuFi-1 cells (**a**) and in NuLi-1 cells (**b**). The iPA-induced effect on chemokines release was reverted by pretreating CuFi-1 and NuLi-1 cells with the ADK inhibitor 5-iodotubercidin (5-Itu, 30 nM for 30 min before iPA treatment). Data are expressed as mean ± SD of three independent experiments, each done in duplicates.*P < 0.05 and **P < 0.01 vs the corresponding control

CF cells lacking a functional CFTR are characterized by higher production of chemokines able to attract neutrophils and macrophages in the airways [17, 18, 21, 28]; therefore, the activity of iPA was determined in both CuFi-1 and NuLi-1 cells by analyzing IL-8 and RANTES release in the absence of pro-inflammatory mediators and after TNFα stimulation using two concentrations of iPA (1.0 and 2.5 µM). Results (Fig. 2A) indicated that iPA was able to reduce significantly chemokine production from 1.0 µM and reached the highest effect at 2.5 µM by inhibiting the secretion of IL-8 (50%) and RANTES (32%) in CF cells stimulated with TNFα. Interestingly, a dose-dependent inhibition of IL-8 (until 23% at 2.5 µM) and RANTES (until 21% at 2.5 µM) was also observed in resting cells. The results obtained in NuLi-1 cells (Fig. 2B), also highlighted a significant iPA-mediated reduction of IL-8 and RANTES secretion by an average of 33% and 63%, respectively, at 2.5 µM especially in TNFα-treated cells. It appears that in normal cells iPA is most effective in reducing RANTES production compared to CuFi-1 cells and its inhibitory effect is higher in the presence of pro-inflammatory stimuli. Of note, the effect of iPA is highly specific since the treatment of both cell lines with 5-Itu, an ADK inhibitor that was reported to phosphorylate and activate iPA inside cells [12], completely reverted the inhibitory effects of iPA in both CF and non-CF cells. Altogether, these results indicate that iPA decreases IL-8 and RANTES release and this effect is particularly elevated in the presence of inflammatory response or in steady state condition of reactive cells as in the case of CF cells exhibiting higher basal levels of both proteins.

### NFκB and STAT3 signaling pathways are differently regulated by iPA in CF and non-CF cells

**Fig. 3:**
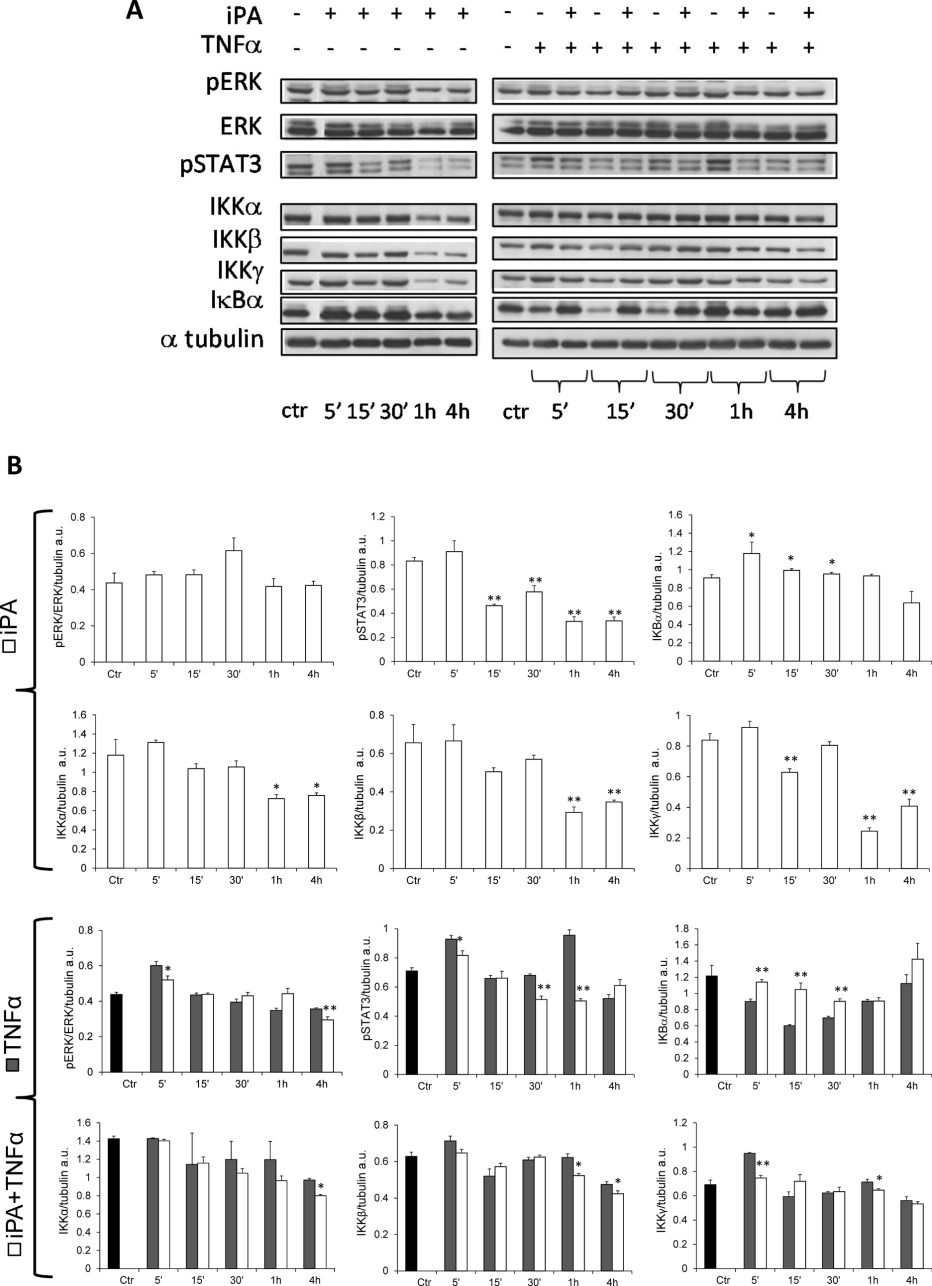
Effects of iPA on MAPK/ERK, STAT3 and NFκB pathways in TNFα-stimulated and -unstimulated CuFi-1 cells. Cells were treated (+) or not (−) with 2.5 µM iPA in presence (+) or absence (−) of TNFα for the indicated time points **A**: Representative western blots on protein extracts showing the effects of iPA on pERK, pSTAT3 and key cell signaling proteins of the NFκB pathway compared to untreated control cells (ctr, time zero). Tubulin was used as loading control. **b** Densitometric analysis showing relative band intensity means (arbitrary units, a.u.) ± SD of three independent experiments. Statistical analysis of iPA data: *P < 0.05 and **P < 0.01 vs the corresponding control

NFκB, MAPK/ERK, and STAT3 pathways plays a central role in the inflammatory signaling cascades leading to cytokine and chemokine production and some studies have demonstrated that NFκB and MAPK/ERK pathways are intrinsically overactivated in various CF cell lines [22, 29, 30]; therefore, to assess whether the effects of iPA could be a result of the modulation of these pathways, CuFi-1 cells were incubated with iPA 2.5 µM and then the main molecular targets of MAPK/ERK, NFκB and STAT3 pathways were analyzed. As shown in Fig. 3A, iPA caused a reduction of ERK phosphorylation only at 5 min and 4 h of treatment in CuFi-1 cells under TNFα stimulation, while no significant changes were observed at all other time points and in the absence of TNFα (Fig. 3B), suggesting that MAPK/ERK pathway can be slightly modulated by iPA in the presence of TNFα-induced inflammation and is not preferentially involved in iPA-mediated reduction of chemokine secretion in the presence of the innate hyper-inflammatory status of CuFi-1 cells. On the contrary, a relevant reduction of STAT3 activity was found either in TNFα-stimulated CF cells at 5 min and from 30 min to 1 h of iPA treatment (Fig. 3A, B), or in CF cells treated with iPA from 15 min to 4 h (Fig. 3B). In the same experimental conditions, the treatment with iPA also negatively affected the background protein expression levels of the NFκB-regulatory subunits IKKα and IKKβ at a longer time exposure (from 1 to 4 h, Fig. 3b), whereas IKKγ subunit was inhibited at 5 min and 1 h (Fig. 3). As concerns the direct NFκB inhibitor IκBα, we also observed a marked and statistical significant increase of its expression starting from 5 min and until 30 min (Fig. 3a) in both TNFα-stimulated or -unstimulated CuFi-1 cells (Fig. 3a, b), thus proposing that iPA, by increasing IκBα expression and down-regulating IKKs subunits is able to inhibit NFκB pathway.

**Fig. 4:**
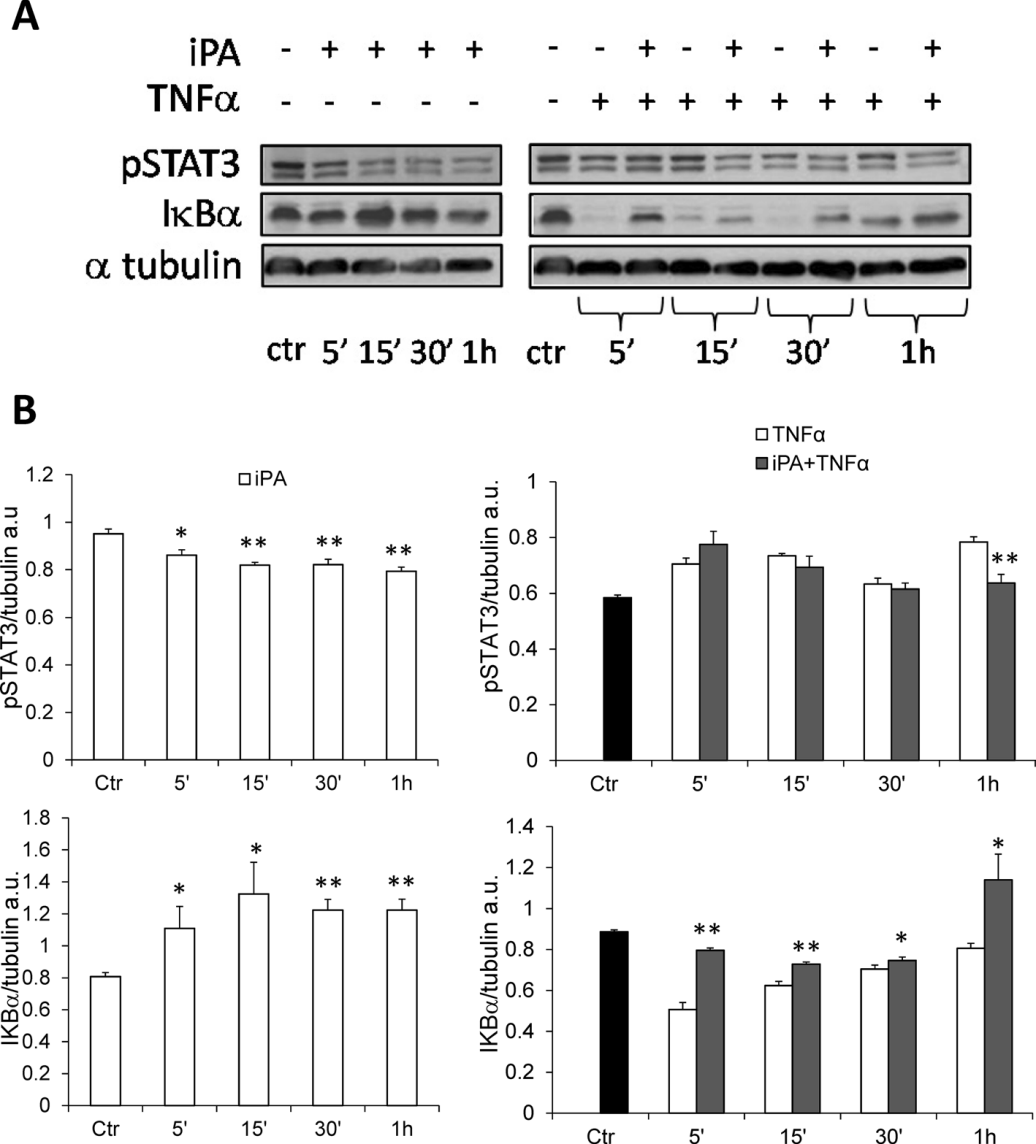
Effects of iPA on pSTAT3 and IκBα in TNFα-stimulated and -unstimulated NuLi-1 cells. Cells were treated (+) or not (−) with 2.5 µM iPA in presence (+) or absence (−) of TNFα for the indicated time points. **a** Representative Western blots showing the effects of iPA on pSTAT3 and IκBα compared to untreated control cells (ctr, time zero). Tubulin was used as loading control. **b** Densitometric analysis showing relative band intensity means (arbitrary units, a.u.) ± SD of three independent experiments. Statistical analysis of iPA data: *P < 0.05 and **P < 0.01 vs the corresponding control

To assess whether a similar mechanism of action was responsible for the iPA-induced effects in normal human bronchial epithelial cells, NuLi-1 were stimulated with TNFα and treated with iPA as in CuFi-1 cells, and analyzed for the expression of proteins whose expression was found markedly modulated in CF cells. Results shown in Fig. 4 indicate that similarly to CF cells, iPA reduced the phoshorylation of STAT3 in the absence of any pro-inflammatory stimulus starting from 5 min and for all over the treatment time. However, we did not observe significant changes in STAT3 activation following iPA treatment in TNFα-stimulated NuLi-1 cells except for 4 h of treatment. Moreover, as in CuFi-1 cells, iPA increased significantly IκBα expression (Fig. 4b) at all time points both in the presence or absence of TNFα treatment, thus indicating that iPA can act by negative modulating key inflammatory proteins involved in the NFκB and STAT3 pathways, even though the iPA-induced effect is quite different in CF and non-CF cells.

### iPA reduces NFκB activity and IL-8 production in HEK/293T cells

**Fig. 5:**
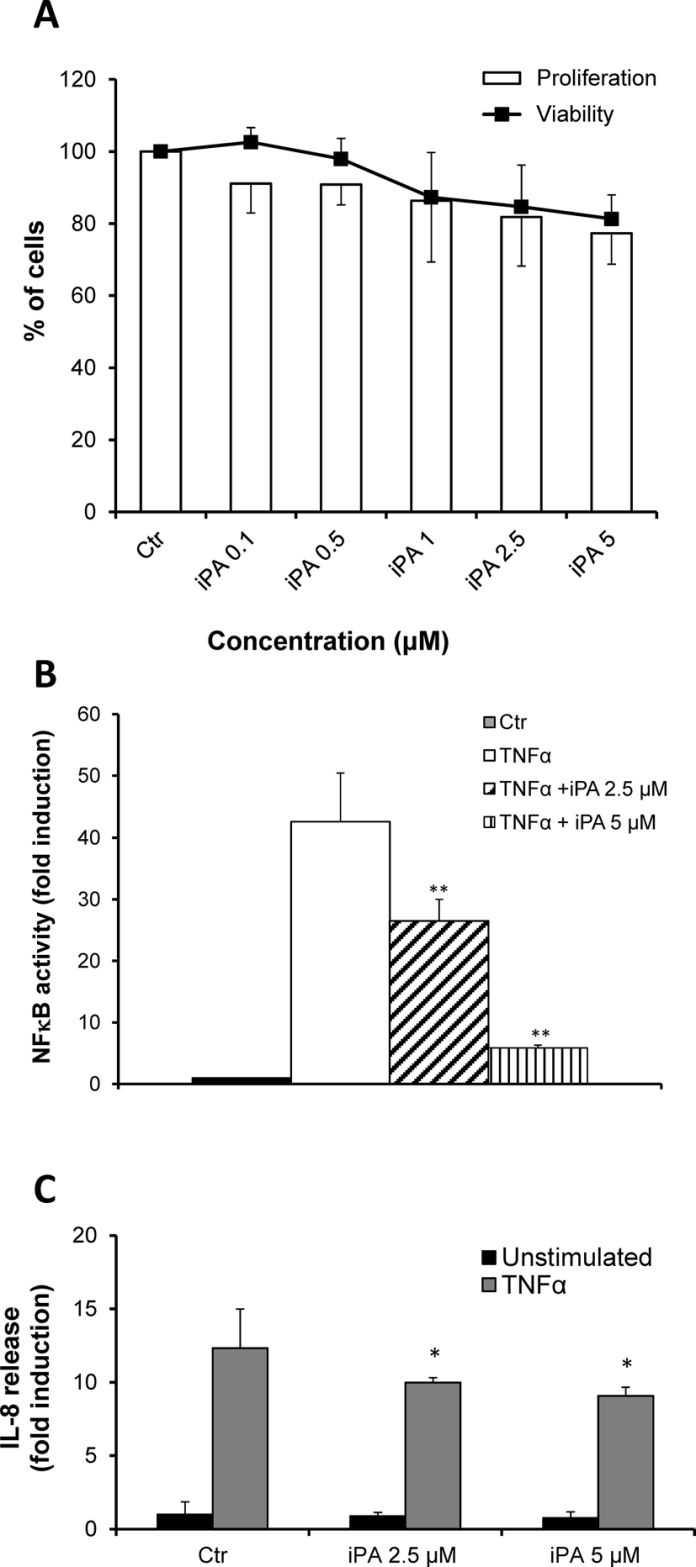
Effects of iPA on HEK293/T cells. **a** Cells were treated with increasing concentrations of iPA for 24 h. Cell viability and proliferation were determined by MTT and BrdU assays and are expressed as percent of controls (100%). **b** HEK293/T cells were transiently transfected with a NFκB reporter plasmid and an internal control plasmid TK-Renilla. After 24 h, cells were preincubated for 1 h with iPA and treated or not (control, ctr) with TNFα for additional 4 h to activate NFκB. Results are expressed as fold activation of NFκB with respect to untreated cells. **c** Cells were treated with the indicated concentrations of iPA and then stimulated or not with TNFα as above. IL-8 production was detected as described in Materials and Methods. Results are all reported as mean ± SD of three independent experiments, each done at least in duplicates. *P < 0.05 and **P < 0.01 vs control

The data here presented have underlined that iPA can act as an anti-inflammatory drug in bronchial epithelial cells regulating particularly the NFκB pathway. To further investigate this effect, we analyzed the effects of iPA treatment on NFκB transcriptional activity. We used HEK/293T cells transiently transfected with a NFκB reporter plasmid, having five tandem copies of the human NFκB binding site fused to luciferase gene [31]. We chose to study the effects of iPA at sub-toxic concentrations, thus we carried out experiments to determine whether iPA could have a cytotoxic effect in the concentration range used in CF and non-CF cells. Results clearly show that there was not any inhibition of both cell proliferating ability and viability at all concentrations tested and for a time-treatment of 24 h (Fig. 5a). On the basis of these results, we analyzed NFκB activity after 6 h of treatment with iPA in the presence or not of TNFα stimulation. Results indicate that iPA was able to reduce drastically the TNFα-induced activation of NFκB in a concentration-dependent manner (Fig. 5b). As expected, this effect was also associated to a significant reduction of IL-8 in the same cell line (Fig. 5c). The inhibitory trend of this cytokine release was comparable to NFκB-dependent transcription activation and was particularly evident in cells stimulated by TNFα (Fig. 5). Altogether, these data corroborate our hypothesis that iPA could exert its anti-inflammatory effect through inhibiting NFκB activity and this leads to the reduction of IL-8.

### iPA positively modulates GPX1 and TR1 expression in CF cells

**Fig. 6:**
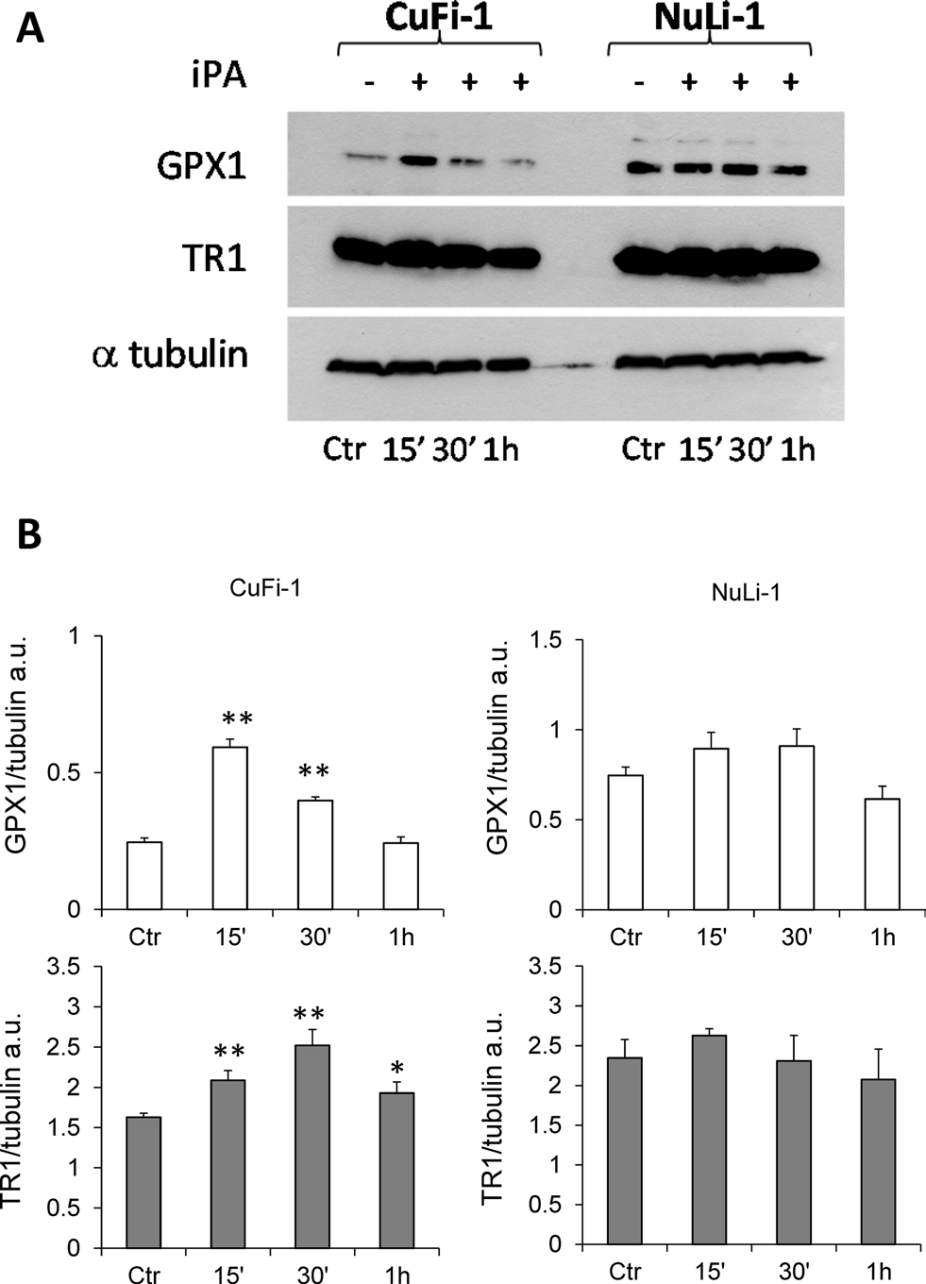
Effects of iPA on Glutathione peroxidase 1 (GPX1) and thioredoxin reductase 1 (TR1) expression in CuFi-1 and NuLi-1 cells. **a** Cells were treated (+) or not (−) with 2.5 µM iPA for the indicated time points and representative Western blots are showed compared to untreated control cells (ctr). Tubulin was used as loading control. **b** Densitometric analysis showing relative band intensity means (arbitrary units, a.u.) ± SD of three independent experiments. *P < 0.05 and **P < 0.01 vs the corresponding control

iPA, as an intermediate of the isoprenoid pathway, plays a crucial role in protein translation, and its lacking can impair selenoprotein synthesis including GPX1 and TR1 which are proteins involved in cell oxidative stress response and anti-inflammatory process [6, 7, 23, 24]. Therefore, we finally investigated whether iPA could affect protein expression levels of GPX1 and TR1. By comparing iPA-mediated effects in the two cited cell lines, we observed that iPA strikingly increased the expression of GPX1 and TR1 in CuFi-1 cells characterized by an intrinsic inflammation also in the absence of any infection (Fig. 6). The GPX1 increase starts from 15 min of iPA treatment and persists until 30 min, while TR1 induction was retained until 1 h. In contrast, in non-CF cells, we showed only a slight but not statistical significant enhancement of GPX1 and no variations in TR1 expression were found. Of note, GPX1 background level of CuFi-1 cells was lower than its normal counterpart NuLi-1, thus suggesting that CFTR dysfunction can affect GPX1 levels and that iPA can partially compensate for this decrease by promoting GPX1 expression.

## Discussion

Findings of our study show that iPA inhibits the inflammatory response reducing IL-8 and RANTES production through a mechanism of action involving primarily the downregulation of the NFκB and STAT3 pathways in a CF cell model characterized by exacerbated inflammation. Despite the increasing literature on the anti-cancer effects of iPA in a broad variety of tumors, very little is known about the possibility that iPA could modulate inflammatory process. Some data suggested an anti-inflammatory activity of the compound in a murine model of croton oil-induced mouse edema [14] and in a Car-S mouse model of 12-O-tetradecanoylphorbol-13-acetate (TPA)-induced oxidative stress [32] but in both cases authors did not provide insights about the underlying mechanism of action. Previous studies from our group also showed that iPA can exert some immunomodulatory properties in IL-2 activated human primary natural killer (NK) cells [13], however no data are available on its anti-inflammatory action in CF disease. To investigate this issue, we analyzed the effect of iPA on IL-8 and RANTES release in CuFi-1 (CFTR^ΔF508/ΔF508^) and NuLi-1 cell lines (CFTR wild type) which are both telomerase-immortalized airway cells, characterized by the ability to constitute a polarized monolayer with a transepithelial activity that mimics the behavior of CF airway epithelial cells in vivo [20]. CuFi-1 cells show overexpression of a number of signal transduction pathways including MAPK/ERK and NFκB that are responsible for the overactivation of several inflammatory and oxidative stress genes such as IL-6, IL-8 and RANTES [33, 34]. The use of these two cell systems allowed us to ascertain whether the effect of iPA was related to the CFTR mutation or was a common anti-inflammatory effect. Indeed, by treating both CF and non-CF cells with iPA, we found that iPA was highly efficacious in reducing IL-8 and RANTES secretion in both CuFi-1 and NuLi-1 cells but to a different extent. iPA was most efficacious in reducing IL-8 in CuFi-1 cells, whereas a greater decrease of RANTES secretion was observed in TNFα-stimulated NuLi-1 cells. A possible explanation for this selective inhibition is that signaling transduction pathways may have altered functionality in primary CF cells compared to normal cells thus contributing differently in reducing chemokine secretion. In fact, chemokines require activation of NFκB pathway but it has been shown that RANTES and IL-8 gene expression are differentially regulated by NFκB in CF vs normal epithelia [35–38]. Di Mango et al. [36] proposed that increased levels of IL-8 secreted by CF IB3 cells may be a result of greater amounts of endogenous nuclear NFκB found in these cells compared to CFTR-corrected cells. Moreover, we demonstrated that iPA was also able to slow down the NFκB pathway by increasing its direct inhibitor IκBα in both CF and TNFα-stimulated non-CF cells, thus suggesting that iPA can act as a NFκB inhibitor decreasing the production of the pro-inflammatory chemokines IL-8 and RANTES. From this point of view, our results may have therapeutic implications since IL-8 is the most abundant chemokine found in broncoalveolar fluid of CF patients [39]. It has been shown that pro-inflammatory genes, including IL-8 and RANTES, are activated following infection with *Pseudomonas aeruginosa* in CF lung [40, 41] substantially contributing to the hyper-inflammatory status of the disease. Even though we do not provide direct evidence on NFκB expression and distribution inside cells, our data indicating increased levels of IκBα following the treatment with iPA suggest that NFκB could be retained in the cytoplasm through its association with IκBα being not allowed to translocate into the nucleus and activate genes controlling the inflammatory process. According to this hypothesis, our data indicated a marked reduction of NFκB transcriptional activity after iPA treatment in HEK/293T cells. On the other hand, TNFα we used to induce inflammation has been shown to mediate directly NFκB activation through the phosphorylation and subsequent degradation of IκBα [42].

Beside the primary role of the NFκB pathway in inflammatory response, other signaling transduction pathways, such as MAPK/ERK pathway, are involved in exaggerated cytokine and chemokine release in CF [21, 30]. Unexpectedly, we found that iPA did not cause high variations in ERK phosphorylation in CF cells thus suggesting that other transduction factors could be responsible for iPA-induced effects. Recently, it has been proposed a role for STAT3 in regulating CF-related inflammation since the overproduction of IL-8 and CXCL1 chemokines is partially controlled by ER stress in a STAT3-dependent manner [22]. STAT3 plays a central role in the inflammatory signaling cascades triggered by LPS, INFγ and other cytokines [43, 44]. Binding of cytokines to specific receptors activates phosphorylation of Janus kinase (JAK) receptor family that in turns phoshorylates STAT3 and promotes its translocation into the nucleus, where it binds to specific gene promoter sequences and inhibits the production of cytokines such as endotoxin-inducible expression of TNFα, IL-6, IL-1β, IL-8 and RANTES [43, 44]. Starting from these observations, we investigated STAT3 signaling in CF and non-CF cells evidencing that the TNFα -induced phosphorylation of STAT3 was inhibited by iPA in CFTR-mutated cells whereas in normal cells iPA-mediated activation of STAT3 was reduced in basal conditions but not substantially affected after the treatment with TNFα. This observation could be explained by the fact that background STAT3 levels are higher in CuFi-1 cells compared to NuLi-1 cells, suggesting an overactivation of this pathway in CF cells. Therefore, we speculate that iPA could exert anti-inflammatory activity in CF cells by targeting selectively NFκB and STAT3 pathways which are both altered in these cells compared to normal cells.

In this study, we also show that the block of ADK activity with its inhibitor 5-Itu counteracts the effects of iPA on chemokines secretion clearly indicating that phosphorylation of iPA into iPAMP is essential for its biological activity. This is in agreement with our previously reported data in other physiopathological cell contexts [10, 12] and suggests that iPA, behaving as an AMP mimetic in CF and non-CF epithelial cells, could activate the adenosine monophosphate (AMP)-activated protein kinase (AMPK) which plays a central role in the regulation of inflammatory response including pulmonary emphysema [45–47]. Of note, AMP-activated kinase (AMPK) is also an ubiquitous metabolic sensor that inhibits CFTR and as highlighted by Hallows et al. [48], the pharmacologic AMPK activation inhibited inflammatory mediator secretion in both wild type- and ΔF508-expressing cells, thus suggesting a iPA-mediated molecular mechanism that needs further investigations. Notably, iPA is present in human tissues where it interferes with cell metabolism affecting cell survival, proliferation and apoptosis by modulating the isoprenoid pathway—a metabolic pathway leading to cholesterol biosynthesis—referred to is itself a derivative [3, 10]. Many alterations of CF cell signaling including decreased expression of nitric oxide synthase 2 (NOS2), reduced function of signal transducer and activator of transcription 1 (STAT1) and reduced function of Rab GTPase have been directly attributed to perturbed cholesterol homeostasis [49], thus it is also possible that intracellular increased levels of iPA could improve these signaling abnormalities. However, it is unlikely that the anti-inflammatory effect of iPA could be ascribable to the regulation of the mevalonate pathway, since the pre-treatment of CuFi-1 cells with mevalonate or lovastatin, which is a HMGCoA reductase inhibitor, failed to revert or interfere with iPA-induced effect on chemokine production (data not shown). Finally, iPA plays a crucial role in protein translation, since its lacking can impair selenocysteine RNA maturation and then selenoprotein synthesis [6, 7]. Consistent with this, we showed that iPA is highly efficacious at inducing the expression of anti-oxidant GPX1 and TR1 selenoproteins in CF cells, suggesting that iPA could exert its anti-inflammatory effect also by maintaining adequate expression levels of selenoproteins. Our findings are in agreement with Dassano et al. [32] who showed that in breast cancer and HL-60 cells induced to differentiate in neutrophilic lineage, iPA was able to trigger the NRF-mediated oxidative response through the induction of gene encoding detoxifying and anti-oxidant enzymes—such as heme oxygenase-1 gene and glutamate-cysteine ligase (GCLC)—that protect cells against ROS and reactive metabolites. In conclusion, as airway epithelial cells contribute significantly to airway inflammation in patients with CF [34, 36]. The fine tuning of cyto-chemokines secretion by iPA along with its ability to improve selenoprotein expression might be an attractive therapeutic approach to reduce excessive airway inflammation which is a major cause of CF morbidity.

## Abbreviations

iPA: N6-isopentenyladenosine
Sec-tRNA: Selenocysteine transfer RNA
5-Itu: 5-Iodotubercidin
MMC: Mitomycin C
ADK: Adenosine kinase
AMPK: AMP-activated protein kinase
tRNA-IPT: tRNA-isopentenyltransferase
DMAPP: Dimethylallyl pyrophosphate
iPAMP: 5′-iPA-monophosphate
GPX1: Glutathione peroxidase 1
TR1: Thioredoxin reductase 1
CFTR: Cystic fibrosis transmembrane conductance regulator
NFκB: Nuclear factor-κB
STAT: Signal transducer and activator of transcription
IκBα: NFκB inhibitorα
JAK: Janus protein tyrosine kinase
ROS: Reactive oxygen species
